# Impact of Frailty on Dietary Habits among Community-Dwelling Older Persons during the COVID-19 Pandemic in Japan

**DOI:** 10.14283/jfa.2021.45

**Published:** 2021-11-23

**Authors:** K. Kinoshita, S. Satake, H. Arai

**Affiliations:** 1 Department of Frailty Research, Center for Gerontology and Social Science, Research Institute, National Center for Geriatrics and Gerontology, 7-430, Moriokacho, Aichi, 474-8511 Japan; 2 Department of Geriatric Medicine, Hospital, National Center for Geriatrics and Gerontology, Obu, Japan; 3 National Center for Geriatrics and Gerontology, Aichi, Japan

**Keywords:** Frail elderly, aged, social isolation, self-isolation, nutrition

## Abstract

**Background:**

The coronavirus disease (COVID-19) pandemic has resulted in reduced physical activity and social interaction. These restrictions may have affected the food intake habits of frail older people more than non-frail older people.

**Objectives:**

To investigate the association between frailty and change in dietary habit during the pandemic.

**Design:**

Cross-sectional mail survey

**Setting:**

Community-based

**Participants:**

The study questionnaire was mailed to 4,436 older residents of Higashiura, Aich Japan, who were aged ≥75 years and who did not need care as of April 1, 2020. Of these, 2,738 participants provided complete answers to the questionnaires (75–96 years old, 49.3% males).

**Measurements:**

The participants’ frailty status and changes in food consumption during social isolation were assessed. Frailty status was assessed using the five-item frailty screening index (i.e., weight loss, low physical function, low physical activity, cognition, and exhaustion). Any participant who reported an increase or a decrease in ≥1 of the 12 food categories was defined as having change in dietary habit. Using multivariate logistic regression analysis, the odds ratios (ORs) and 95% confidence intervals (CIs) of frailty for changes in diet were estimated by adjusting for age, sex, BMI, and living alone. In each of the 12 food categories, the proportion of participants with increased and decreased food intake was compared between the groups.

**Results:**

Among the participants, 470 (17.2%) were frail, and 1,097 (40.1%) experienced a change in dietary habit under social restriction. The adjusted OR (95% CI) of the frail group for a change in dietary habit was 2.01 (1.63–2.47, p<0.001). Participants with decreased consumption of meat, fish, seaweed and mushroom, and fruits and those with increased consumption of eggs, bread, and noodles tended to be frail.

**Conclusion:**

The nutritional intervention for frail older people should be strengthened during the pandemic.

## Introduction

Coronavirus disease (COVID-19) has spread rapidly, and the number of deaths has increased worldwide. The lockdown measure was instated as one of the means of COVID-19 prevention in several nations, including Japan. Many geriatricians are concerned about the negative impact of the lockdown on various health aspects in older adults, such as loneliness, physical activity, etc. (1–3). Loneliness could be related to physical disability and mortality (4–6). Low physical activity during social isolation may induce muscle weakness and physical impairment. In fact, a Japanese community-based study revealed that the incidence of frailty one year after the COVID-19 pandemic in older adults living alone and having few social activities was twice the incidence among older adults who were not living alone and had many social activities (7). Importantly, self-isolation during the pandemic also changed people’s lifestyle behaviors that affected dietary intake through food shopping and economic status. It is reported that reduced food availability and poor economic status were related to a lower diet quality, which is a key factor for the poor health status of the older population (3, 8–10).

Social support plays a role in maintaining a good health status of frail older people (11, 12). A previous study suggested that a neighbor relationship improved poor diet quality among adults who live in communities with low food availability (13). Although social isolation during the COVID-19 pandemic has kept away these social supports from older adults, there is no study on the association between frailty status and food consumption during self-isolation in the pandemic in older populations. In particular, it is important to target older people over the age of 75 due to a significantly higher prevalence of frailty (14).

Social preventive measures of COVID-19 may lead to altered health conditions of older adults in future. Therefore, investigating and characterizing the impact of frailty status on dietary changes during self-isolation in pandemics is important for considering nutrition support and care. In the present study, we hypothesized that frail older people are susceptible to poor food intake; therefore, we investigated the association between frailty and changes in food intake during the COVID-19 pandemic in the community.

## Material and methods

### Study design and participants

This was a cross-sectional mail survey of older residents aged ≥75 years who did not need care and lived in Higashiura, Aich, Japan as of April 1, 2020. Higashiura is a town with a total surface area of 31.14 km^2^ and an estimated population of 50,154 in 20,719 households as of March 31, 2020 (15). Of these, 12,763 (25.4%) residents were aged ≥65 years, and 6,420 (12.8%) were aged ≥75 years (15). In Japan, the government announced the first state of emergency and a going-out restriction between April 10, 2020 and May 14, 2020. This restriction included Higashiura residents.

We sent a questionnaire to older residents who were not certified as requiring care or support on July 1, 2020 to investigate their frailty status and life style changes, such as food intake during the first social isolation in the COVID-19 pandemic. We asked them to return it by August 31, 2020. It takes ∼15 minutes to complete the questionnaire.

Participants were able to allow their family members to fill in the questionnaire on their behalf, without a record of surrogate respondents. Of the 4,436 participants, 3,187 (71.8%) responded to the questionnaires and returned them. Participants who returned incomplete responses (n=449) were excluded, and the data of 2,738 participants were analyzed (males n=1,349, 49.3%).

This study was approved by the Ethics Committee of Human Research of the National Center for Geriatrics and Gerontology, Japan (No. 1463), and was conducted in accordance with the Declaration of Helsinki. Informed consent was obtained using the opt-out method approved by the Personal Information Protection Commission.

### Definition of frailty

Frailty status was defined using the frailty screening index (FSI) (16), which was based on the Cardiovascular Health Study criteria (17) and Kihon checklist (18, 19). The FSI has five yes/no questions on weight loss, low physical function, low physical activity, cognition, and exhaustion. The questions and scoring are as follows: “Have you lost 2 kg or more in the past 6 months?” Yes=1, “Do you think you walk slower than before?” Yes=1, “Do you go for a walk for your health at least once a week?” No=1, “Can you recall what happened 5 minutes ago?” No=1, and “In the past 2 weeks, have you felt tired without a reason?” Yes=1. The score ranges from 0 to 5. Participants with a score ≥3 points were considered to be frail. The FSI was developed as a self-reported assessment tool for defining frailty, and was validated as a predictor of disability and death by mailing survey (16).

### Assessment of dietary changes during social isolation

Dietary changes were assessed by increase/decrease in the intake of 12 food categories: (1) meat, (2) fish, (3) egg, (4) soy and soy products, (5) milk and dairy, (6) vegetables, (7) seaweed and mushroom, (8) fruits, (9) rice, (10) bread, (11) noodles, and (12) alcohol, confectionery, and soft drinks. The following questions were asked for each food category: “How did your XXX (e.g., meat) intake change during the social restriction period due to a state of emergency?”. The answers were: “(1) increased, (2) decreased, or (3) no change” Any participants who reported an increase or decrease in one or more of the 12 food categories was defined as having dietary change during social isolation.

### Other life-style factors changed in social isolation

Lifestyle factors such as food shopping frequency, food costs, supplementation, going-out for physical activity, going-out for friendship exchange, and speaking conversation frequency were assessed. The questions in this regard were as follows: “Did you reduce the frequency of your food shopping during social restriction due to a state of emergency? Yes/no,” “Did you save on food costs because of economic problems during social restriction due to a state of emergency? Yes/no,” “Did you start nutritional supplementation during social restriction due to a state of emergency? Yes/no,” “Did you go out for physical activities such as walking and farm work during social restriction due to a state of emergency? Yes/no,” “Did you go out for friendship exchange during social restriction due to a state of emergency? Yes/no,” and “Did you increase the days of not talking to anyone all day long (including telephone conversation) during social restriction due to a state of emergency? Yes/no.”

### Covariates

Age, sex, body mass index (BMI), living alone, and solitary eating were assessed. Age and sex were obtained from the administrative data from Higashiura. BMI was calculated using the participant’s self-reported height and weight. Solitary eating was assessed using the question “Do you eat with someone at least once a day? Yes/no.”

### Statistical analysis

The means and standard deviations (SDs) for continuous variables and the number and percentage (%) for categorical variables were calculated. The t-test or chi-square test was used to compare participants’ characteristics between the frail and non-frail groups. The chi-square test or Fisher’s exact test was used to compare the proportion of participants who had increased or decreased intake in each of the 12 food categories between the frail and non-frail groups.

To examine the association between frailty status and dietary change, multiple logistic regression analysis was performed. The odds ratio (OR) with 95% confidence interval (CI) of the frail group for dietary change was estimated using the non-frail group as a reference, after adjusting for age, sex, BMI, and living alone.

All statistical analyses were performed using IBM SPSS Statistics ver. 27.0 (IBM Japan, Tokyo, Japan), and statistical significance was indicated by two-sided P values <0.05.

## Results

**Figure 1:**
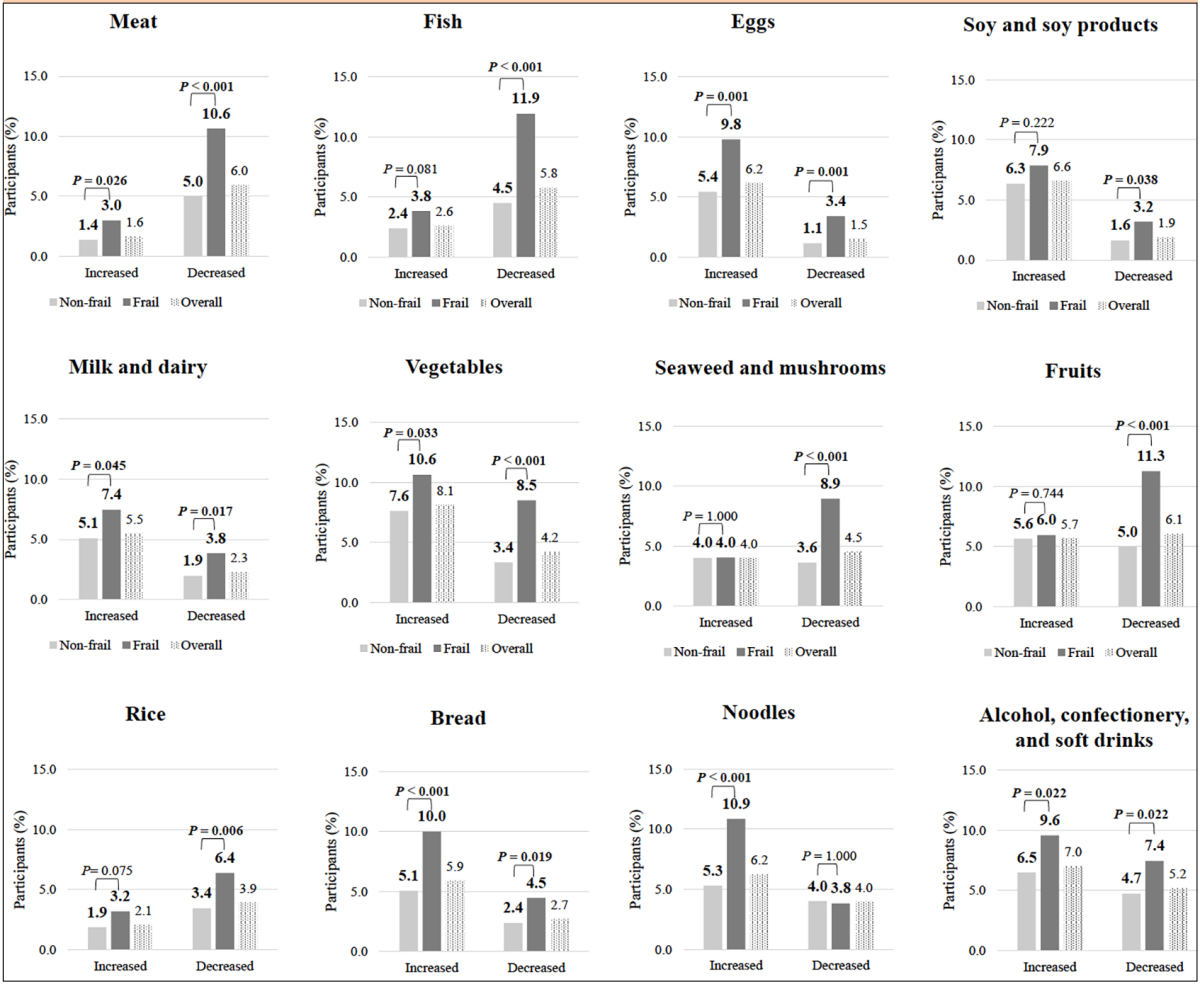
Differences in the 12 food categories with increased/decreased intake during social isolation in a pandemic between the frail and non-frail groups

The mean ± SD (range) of age was 79.8 ± 3.8 (75–96) years. Among the 2,738 participants, 470 (17.2%) were frail, and 1,097 (40.1%) experienced dietary changes. The participants’ characteristics are shown in Table 1. The frail group was significantly older (higher age), shorter (lesser height), and had a larger proportion of females than the non-frail group. Lifestyle changes during social isolation are shown in Table 2. Dietary change was more common in the frail group than in the non-frail group (53.6% vs. 37.3%, P <0.001). The frail group had a significantly higher proportion of changed lifestyles than the non-frail group: reduction in food shopping frequency (72.6% vs. 6.8%, P =0.006), taking nutritional supplementation (7.7% vs. 3.2%, P <0.001), reduction in physical activity such as walking and farm work (52.3% vs. 36.1%, P <0.001), and increase in the number of days of not talking to anyone all day long (26.4% vs. 17.1%, P <0.001). Figure 1 shows the difference in the 12 food categories with increased or decreased intake between the frail and non-frail groups. Overall, we found more older adults who decreased their intake of meat, fish, and rice as opposed to those who increased their intake of these foods. Additionally, participants who increased their intake of egg, soy and soy products, milk and dairy, vegetables, bread, noodles, alcohol, confectionery, and soft drink were greater than those who decreased their intake of these foods. On comparing the frail and non-frail groups, those who consumed less meat, fish, seaweed and mushrooms, and fruits were more frequently frail than non-frail (frail group vs. non-frail group: meat, 10.6% vs. 5.0%, P <0.001; fish, 11.9% vs. 4.5%, P <0.001; seaweed and mushrooms, 8.9% vs. 3.6%, P <0.001; and fruits, 11.3% vs. 5.0%, P <0.001); whereas, those who consumed more egg, bread, and noodles were more likely to be in the frail group than in the non-frail group (frail group vs. non-frail group: egg, 9.8% vs 5.4%, P =0.001; bread, 10.0% vs 5.1%, P <0.001; and noodles, 10.9% vs 5.3%, P <0.001).

**Table 1:** Participants’ characteristics

	**Frail**	**Non-frail**	**P value**
	**n =470**	**n =2268**	
Males	209 (44.5)	1140 (50.3)	0.023
Age, years	81.2 ± 4.5	79.8 ± 3.8	<0.001
Height, cm	155.0 ± 8.8	156.5 ± 8.8	0.001
Weight, kg	55.3 ± 11.3	55.8 ± 9.7	0.299
BMI, kg/m^2^	22.9 ± 3.5	22.7 ± 3.1	0.336
Living alone	67 (14.3)	361 (15.9)	0.402

The values are presented as mean ± SD or n (%); P values were obtained using the t-test for continuous variables and the chi-square test for categorical variables; BMI, body mass index; SD, standard deviation.

**Table 2:** Lifestyle changes during social isolation in the COVID-19 pandemic

	**Frail n =470**	**Non-frail n =2268**	**P value**
Change in dietary habit	252 (53.6)	845 (37.3)	<0.001
Reduced frequency of food shopping	341 (72.6)	1514 (66.8)	0.006
Saved on food costs	52 (11.1)	236 (10.4)	0.680
Started nutritional supplementation	36 (7.7)	72 (3.2)	<0.001
Reduced physical activity such as walking and farm work	246 (52.3)	819 (36.1)	<0.001
Reduced going out for friendship exchange	314 (66.8)	1427 (62.9)	0.096
Increased days of not talking to anyone all day long (including telephone)	124 (26.4)	388 (17.1)	<0.001

The values are presented as n (%); P values were obtained using the chi-square test.

The bar graphs show the proportion of participants who increased or decreased their intake of 12 food categories in the frail and non-frail groups. The dark color bars are for the frail group. The light color bars are for the non-frail group. The dot bars are for the overall participants. P values were obtained using the chi-square test or Fisher’s exact test to compare between the frail and non-frail groups.

Table 3 shows the multivariable-adjusted associations between frailty and change in dietary habit during social isolation. After adjusting for age, sex, BMI, and living alone, the adjusted OR (95% CI) of the frail group for dietary change was 2.01 (1.63–2.47), using the non-frail group as reference.

**Table 3:** Multivariable-adjusted associations between frailty and change in dietary habit during social isolation

	**Crude**			**Adjusted**		
	**OR**	**95% CI**	**P value**	**OR**	**95% CI**	**P value**
Frail	1.95	1.59–2.38	<0.001	2.01	1.63–2.47	<0.001
Age				0.99	0.97–1.01	0.160
Male				0.72	0.61–0.85	<0.001
BMI				1.03	1.01–1.06	0.015
Living alone				2.23	1.80–2.77	<0.001

Multivariable logistic regression models were used to estimate ORs and 95% CIs; BMI, body mass index; OR, odds ratio; CI, confidence interval.

## Discussion

In summary, we found that the dietary habits of frail older people were more strongly affected by social isolation during the COVID-19 pandemic than those of non-frail older people. In this study, 470 (17.2%) participants were frail older adults, and 1,097 (40.1%) participants experienced dietary changes. There was a positive association between frailty and change in dietary habits — after adjusting for covariates such as living alone, the OR of frailty for dietary changes was 2.01 (relative to the non-frailty group). In addition, we found more participants who decreased their consumption of meat, fish, seaweed and mushrooms, and fruits and increased their consumption of eggs, bread, and noodles in the frail group than in the non-frail group. To the best of our knowledge, this is the first study to investigate the association between frailty and change in dietary habits during the social isolation of the COVID-19 pandemic among older residents over 75 years old who did not require care. This study data from approximately 3,000 participants is valuable because previous studies were online surveys and assessed only a small number of older people (20, 21).

In this study, the number of people who had reduced food shopping times during social isolation was significantly higher among frail older adults than among the non-frail ones. A previous study reported that older adults who went to the supermarket ≥3 times per week consumed more meat than those who went <3 times (22). Older people with reduced food availability have a poorer diet quality and lower intake of vegetables and fruits than those who have easy access to food (13, 23). Although social support such as neighbor relationship has been suggested to improve dietary quality (13, 24), self-isolation and restricted social support in the prevention of COVID-19 may have created difficulties in maintaining food availability for frail older people with limited access to transportation. The proportions of participants who increased the number of days of not talking to anyone all day long (including telephone conversation) or reduced their outdoor activities such as walking and farm work during the pandemic was significantly higher in the frail group than in the non-frail group. Daily speaking may play an important role in maintaining oral function (25, 26). Reduced interaction with others has been associated with depression, and people with depression have been shown to have a poorer diet quality than people without depression (27, 28). Physical activity has also been associated with depression and appetite, and physical activity levels may affect food consumption through the mechanisms of appetite control (29, 30).

The intake of some of the 12 food categories decreased, especially in the frail group. Meat and fish are the main source of high-quality proteins and are important for maintaining physical function in older adults (31). Seaweed, mushrooms, and fruits are rich in vitamins, minerals, dietary fiber, prebiotics, and antioxidants. In particular, fruits have health-promoting effects, such as inflammation relief, prevention of chronic disease and disability, and reduction of mortality (32). Conversely, the frail older people consumed bread and noodles more than the non-frail older people. Bread and noodles are easy to eat and have a long shelf life than fresh foods; thus, these foods might have been available for older adults who had difficulty in going to shop during the social isolation period. However, most bread and noodles in Japan are made from refined wheat, which has a high glycemic index. Prolonged intake of these food-biased diets may lead to obesity and impaired glucose tolerance (33). A positive association of the combination of frailty and diabetes with mortality and incidence of disability has been suggested (34). Therefore, these food consumption changes might lead to poor health states of frail older adults in future. On the other hand, some frail people consumed protein-rich foods (i.e., meat, egg, soy and soy products, and milk and dairy) and vegetables more. Furthermore, the proportion of people with newly started nutritional supplementation during the pandemic was higher in the frail group than in the non-frail group. These results indicate that some of the frail older people may have tried to improve dietary habits during the pandemic; however, this could not be clarified in this study. In future studies, it is important to address the impact of these food consumption differences on changes in frailty status.

This study has several limitations. First, total daily intake could not be assessed; thus, whether dietary changes led to excessive or deficient dietary intake was unclear. Second, with the low number of infected people, the lockdown in Japan may have been less strict than it was in other countries. Japanese were ‘restricted’ and not ‘banned’ from going out of their homes; thus, the influence of social isolation on dietary habits in Japanese older people may be less than in other countries. Future studies are needed to investigate the same in different populations as well as to compare the finding in different study periods in Japan. Third, as this was a cross-sectional study, there is a possibility that frailty was a result of dietary changes during the social isolation period in some participants. Individual lifestyle during the COVID-19 pandemic may be affected by factors associated with healthy (i.e., physical activity, good nutrition/diet, sleep, social relations) and unhealthy aging (i.e., depression, cognitive impairment,), which are also associated with frailty (35, 36). Thus, the association between frailty and dietary changes is likely a complex combination of multiple factors that were not assessed in the present study. Fourth, we could not assess participant education levels and their cognitive function objectively. Some participants may have had mild cognitive impairment, although we excluded older adults requiring care or support in the present study.

In conclusion, frail older residents aged ≥75 years were positively associated with change in dietary habit during social isolation in the COVID-19 pandemic. Frail older adults had decreased intake of meats, fish, seaweed and mushrooms, and fruits and had increased intake of eggs, bread, and noodles. These findings may provide important insights for considering social approaches to good dietary habits for frail older residents.

## Notes

*Ethical standards:* This study was carried out in accordance with the ethical standard.
